# Letter to the Editor on ‘Effects of exposure to a mobile phone on testicular function and structure in adult rabbit’ by Salama *et al.*

**DOI:** 10.1111/j.1365-2605.2009.00983.x

**Published:** 2010-02

**Authors:** Alexander Lerchl, Christian Bornkessel

**Affiliations:** * Jacobs University BremenBremen; † IMST GmbH, Kamp-LintfortGermany E-mail: a.lerchl@jacobs-university.de

Dear Editor,

Recently, a paper was published (Salama *et al.*, 2008) describing the effects of electromagnetic fields from a mobile phone on testicular functions in New Zealand White (NZW) rabbits. We have a number of concerns; the two most important ones deal with the exposure conditions and the biological effects respectively. Exposure was carried out by a GSM mobile phone at 800 MHz in ‘standby position’. In standby mode (we assume that this is meant by ‘standby position’), a GSM mobile phone does not transmit RF electromagnetic fields, as in talk mode, except for short signals once in a while (every 0.5 h up to several hours, depending on the network operator) indicating its presence to the base station. Therefore, the animals were not exposed to high-frequency electromagnetic fields for eight hours per day, but only for a few seconds! The reported whole-body SAR values of 0.43 W/kg are therefore probably wrong because we expect they were calculated for the transmit frequency. Besides, GSM 800 is not the standard for mobile communication in Japan (where the experiments were performed). Although the authors did not give any details of how they measured the electric fields, it appears very likely that they used a broadband probe which picks up fields usually between 100 kHz and some GHz. Actually, a measurement of several GSM mobile phones fields in the laboratory of IMST revealed electrical field strengths of approximately 3 V/m directly at the phones’ display. A frequency analysis showed, however, that the emissions were mainly in the kHz range and they originate from the phones’ internal electronics (e.g. display processor, switching power supply).

The other point of concern relates to the reported values for sperm counts (Fig. 2, Salama *et al.*, 2008), and sperm motility (Fig. 3, Salama *et al.*, 2008) respectively, showing (in both control groups and the exposed group until week 7) negligible coefficients of variation (CV; standard deviations divided by the means) of approximately 3%, and only minute changes in the mean values (<4%) during the experimental period of 12 weeks. In other words, both the within- and the between-variability of these parameters are exceptionally small and in sharp contrast to previously published data on NZW rabbits (Williams *et al.*, 1990), showing CVs which are higher by an order of magnitude. Also, the extremely sudden (and thereafter entirely stable) effects on sperm counts and motility respectively are biologically incomprehensible.

We therefore express our severe concerns about the validity of the exposure conditions and the reported biological effects in the study.
